# Identification of the novel therapeutic targets and biomarkers associated of prostate cancer with cancer-associated fibroblasts (CAFs)

**DOI:** 10.3389/fonc.2023.1136835

**Published:** 2023-03-03

**Authors:** Xinyu Zhai, Xinglin Chen, Zhong Wan, Minyao Ge, Yi Ding, Jianyi Gu, Jinjun Hua, Dongdong Guo, Mingyue Tan, Dongliang Xu

**Affiliations:** Urology Centre, Shuguang Hospital Affiliated to Shanghai University of Traditional Chinese Medicine, Shanghai, China

**Keywords:** cancer-associated fibroblasts, prostate cancer, CENPF, PPAR signaling pathway, therapeutic targets

## Abstract

Globally, prostate cancer remains a leading cause of mortality and morbidity despite advances in treatment. Research on prostate cancer has primarily focused on the malignant epithelium, but the tumor microenvironment has recently been recognized as an important factor in the progression of prostate cancer. Cancer-associated fibroblasts (CAFs) play an important role in prostate cancer progression among multiple cell types in the tumor microenvironment. In order to develop new treatments and identify predictive and prognostic biomarkers for CAFs, further research is needed to understand the mechanism of action of prostate cancer and CAF. In this work, we performed the single-cell RNA sequence analysis to obtain the biomarkers for CAFs, and ten genes were finally regarded as the marker genes for CAFs. Based on the ssGSEA algorithm, the prostate cancer cohort was divided into low- and high-CAFs groups. Further analysis revealed that the CAFs-score is associated with many immune-related cells and immune-related pathways. In addition, between the low- and high-CAFs tissues, a total of 127 hub genes were discovered, which is specific in CAFs. After constructing the prognostic prediction model, SLPI, VSIG2, CENPF, SLC7A1, SMC4, and ITPR2 were finally regarded as the key genes in the prognosis of patients with prostate cancer. Each patient was assigned with the risk score as follows: SLPI* 0.000584811158157081 + VSIG2 * -0.01190627068889 + CENPF * -0.317826812875334 + SLC7A1 * -0.0410213995358753 + SMC4 * 0.202544454923637 + ITPR2 * -0.0824652047622673 + TOP2A * 0.140312081524807 + OR51E2 * -0.00136602095885459. The GSVA revealed the biological features of CAFs, many cancer-related pathways, such as the adipocytokine signaling pathway, ERBB signaling pathway, GnRH signaling pathway, insulin signaling pathway, mTOR signaling pathway and PPAR signaling pathway are closely associated with CAFs. As a result of these observations, similar transcriptomics may be involved in the transition from normal fibroblasts to CAFs in adjacent tissues. As one of the biomarkers for CAFs, CENPF can promote the proliferation ability of prostate cancer cells. The overexpress of CENPF could promote the proliferation ability of prostate cancer cells. In conclusion, we discuss the potential prognostic and therapeutic value of CAF-dependent pathways in prostate cancer.

## Introduction

In male reproduction, the prostate is the main accessory gland (1). The glandular epithelium secretes prostate fluid, which contains essential molecules that not only assist the prostate in its function but also contribute to the process of ejaculation (2). Clinically, prostate cancer can be aggressive, with metastatic progression, or asymptomatic, with indolent progression (3). The most common cancer in men and the leading cause of death in the Western world is prostate cancer (4). According to the National Cancer Institute, there will be 174,650 new cases and 31,620 deaths in 2019, which makes it the second-leading cause of cancer-related death among American men (5). There is a wide variation in the natural history of prostate cancer. There will be 1 in 6 American men who develop prostate cancer over the course of their lifetime. Of those, only 1 in 6 will die from prostate cancer, and most of them will not develop any clinical symptoms (6). Patients with prostate cancer can be diagnosed with prostate cancer using serum prostate-specific antigen (PSA). It has, however, been found to lack specificity, which results in overdiagnosis and overtreatment of prostate cancer. A patient’s survival depends on early disease diagnosis and treatment options available for prostate cancer. Androgens regulate this malignancy by signaling through androgen receptors (AR). Thus, androgen deprivation therapy (ADT), the first-line therapy that initially leads to cancer regression, aims to eliminate AR signaling (7). Thus, androgen deprivation therapy (ADT), the first-line therapy that initially leads to cancer regression, aims to eliminate AR signaling (8). It is common for a tumor to develop into metastatic castration-resistant prostate cancer after undergoing hormone therapy, leading to poor survival and few treatment options (9). As the exact mechanism of prostate cancer metastasis remains unclear, scientists are investigating this further.

It is possible for cancer cells as well as the tumor-associated microenvironment to exhibit altered molecular signatures in a given tumor (10). It is clear that all of these changes are accompanied by varying degrees of differentiation between tumor cells, tumor populations, or topologically or anatomically isolated tumors (11). In addition to being major components of tumor microenvironments, cancer-associated fibroblasts (CAFs) play an important role in a range of physiological functions, such as cell differentiation, proliferation, extracellular matrix remodeling, and cell migration (12). Angiogenesis, tumor progression, recurrence, and metastasis are all important factors in tumor biology, including tumorigenesis, tumor growth, energy metabolism, tumor immunity, and tumor immune response (13). There are various intracellular and extracellular factors that mediate the biological activities of CAFs, especially those signaling pathways that may be targets of anticancer therapies (14). Molecular and functional heterogeneity can be observed in CAFs in different types of cancer and even in the different stages of the same type of cancer. As CAFs crosstalk with cancer cells specifically, any therapeutic strategy developed should take advantage of this specificity to maximize drug efficacy (15). It has been previously demonstrated that CAF-derived LOXL2 is an important mediator of intercellular communication in the prostate tumor microenvironment and may be useful as a potential therapeutic target (16). In addition, The GPR30 protein in prostate CAFs regulates CAF-TAM interactions in the TME and provides new insight into prostate cancer therapy through regulation of the TME, according to another study (17). However, the role of CAFs in the progression of prostate cancer has not been fully explored.

In this work, we aim to explore the potential correlation between CAFs and prostate cancer. In addition, we also constructed the prognostic prediction model based on the CAFs-related genes. The key genes involved in the process between CAFs and prostate cancer have also been revealed. Furthermore, the enrichment analysis based on the hub genes involved in the prostate was applied by GO, KEGG, GSEA and GSVA analysis.

## Methods

### Dataset downloaded

The expression data, as well as the clinical characteristics of patients with prostate cancer, was obtained from the TCGA database (The Cancer Genome Atlas, https://portal.gdc.cancer.gov/). TISCH2 (Tumor Immune Single-cell Hub 2, http://tisch.comp-genomics.org/home/) is a scRNA-seq database focusing on tumor microenvironments. With TISCH2, it is possible to analyze the TME across different cancer types based on detailed cell-type annotations at the single-cell level. In this work, the single-cell RNA sequencing data were downloaded from the TISCH2.

### Single sample gene set enrichment analysis

The ssGSEA method was used to quantify the individual scores for each tumor case. ssGSEA computes overexpression measures for a list of genes of interest using a rank-based method. CAFs signatures from previous studies were used to calculate ssGSEA scores.

### Construction of the prognostic prediction model based on the prostate cancer cohort

Subsequently, we performed univariate COX regression analyses for the key gene of CAFs, identifying significantly associated genes with prognosis. With the “glmnet” R package, we used the least absolute shrinkage and selection operator (LASSO) regression and multivariate COX regression to further narrow down candidate CAFs-related prognostic biomarkers. The samples were divided into low-risk and high-risk groups using the “survminer” R package. In addition, the survival analysis was also performed in R.

### Enriched pathway analysis based on the key genes

Analyses of GO function annotations based on the GO database (http://geneontology.org/page/go-database) and of KEGG pathway annotations based on the KEGG database (http://www.kegg.jp/kegg/ko.html) were performed to explore the potential pathways that are closely associated with selected genes. The GO enrichment analysis consists of the biological process (BP), the molecular function (MF), and the cellular component (CC). As part of this study, the enrichment of gene sets was assessed using GSVA, a method that is nonparametric and unsupervised. By scoring gene sets of interest comprehensively, gene-level changes in this analysis are converted to pathway changes, and the biological function of the sample is then determined. A database of molecular signatures was used to retrieve gene sets for this study. The GSVA algorithm was used to assess potential biological functional changes in various samples.

### Differentially expressed analysis

In this study, we used the Limma package of the R language to investigate differences in mRNA expression. In TCGA, false positives were corrected using adjusted P values. False positive results were corrected with adjusted P values in TCGA. As a threshold for mRNA differential expression, P < 0.05 and log2 (fold change) > 1 or log2 (fold change) < -1” were defined.

### Immune cell infiltration

This dataset contains RNAseq data and the corresponding clinical information of prostate cancer tumors derived from The Cancer Genome Atlas (TCGA) (https://portal.gdc.com). Our immune score assessment was done with immunedeconv, an R package integrating algorithms, such as TIMER, xCell, MCP-counter, CIBERSORT and EPIC. Benchmarking has been conducted on these algorithms, and each has been found to have unique performance characteristics and advantages. The results described above were obtained using the R packages ggplot2 and pheatmap (v4.0.3). The expression values of genes related to immune checkpoints were extracted, and the expression of genes related to immune checkpoints was observed.

### Gene set variation analysis

A non-parametric, unsupervised method called GSVA was used for evaluating gene set enrichment. In order to convert gene-level changes into pathway-level changes, we scored the gene set of interest. Following this, we determined the biological function of the sample by retrieving gene sets from the molecular signatures database. In order to assess possible biological changes, we carried out comprehensive GSVA analyses on a variety of samples.

### Cell culture

In our lab, two human prostate cancer cell lines were restored: PC-3, a prostate cancer 3 cell line, and LNCap, an androgen-dependent prostate cancer cell line. The PC-3 and LNCap cells were cultured at 37 °C in 5% CO2. A manufacturer’s instruction was followed for transfecting the expression plasmid.

### Cell counting kit-8 assay

By counting the number of viable cells after various treatments, the viability of cells was measured using the CCK-8 assay. The PC-3 and LnCap cells were plated in 96-well plates at a density of 1000 cells per well for three replications. The absorbance was measured with a microplate reader at 450 nm.

### 5-Ethynyl-2′-deoxyuridine staining

The PC-3 and LNCap cells were stained with EdU using 0.1% EdU added to the culture flasks. The EdU assay was performed with the protocol. The EdU was probed with Apollo staining.

### Statistical analysis

All statistical analyses were performed using R software, and a p-value of less than 0.05 was considered statistically significant on both sides.

## Results

### Identification of the CAFs-related biomarkers in prostate cancer cohort

**Figure 1:**
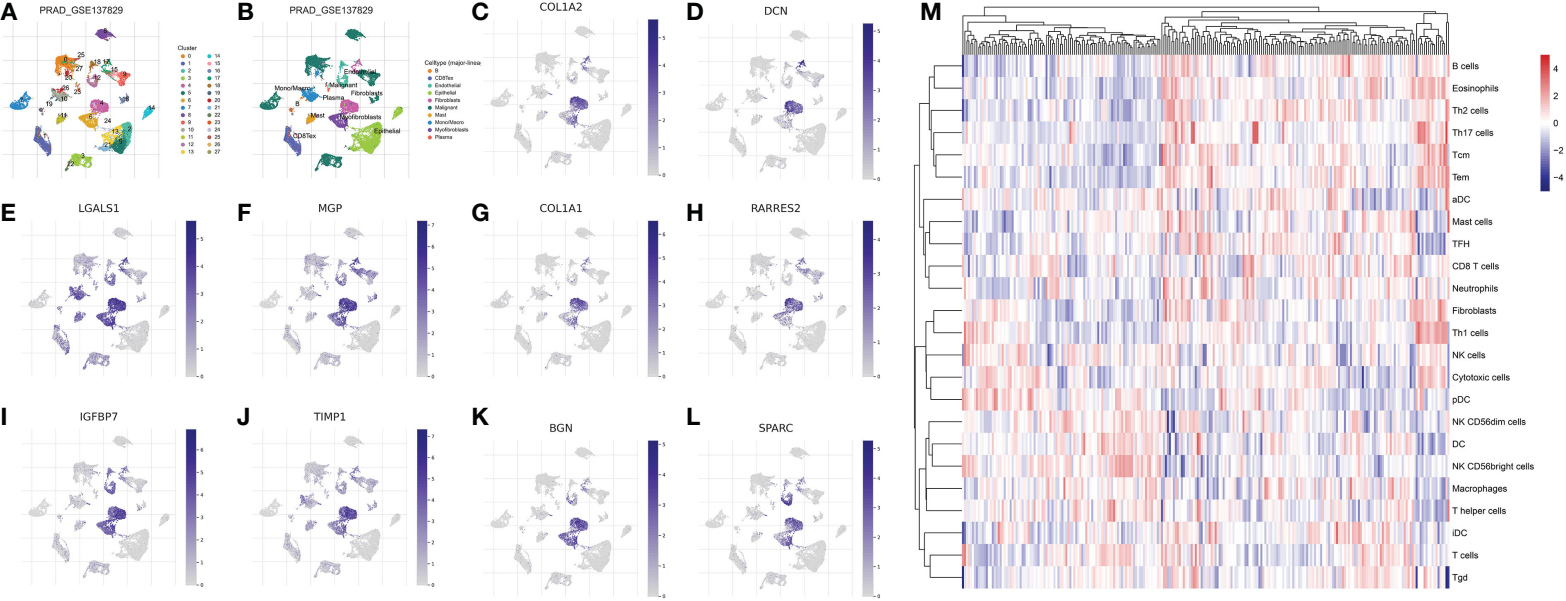
**(A, B)** The single-cell RNA plot of the GSE137829 dataset; The single-cell RNA sequencing analysis of COL1A2 **(C)**; DCN **(D)**; LGALS1 **(E)**; MGP **(F)**; COL1A1 **(G)**; RARRES2 **(H)**; IGFBP7 **(I)**; TIMP7 **(J)**; BGN **(K)**; SPARC **(L)**; **(M)** The results of the ssGSEA analysis.

Firstly, in order to explore the biomarkers for the CAF in the prostate cancer cohort, we performed single-cell RNA sequencing. The dataset of GSE137829 was downloaded from the TISCH2. The single-cell RNA sequencing demonstrated that many types of cells are involved in the prostate cancer cells, such as B cells, CD8 T cells, endothelial cells, epithelial cells, fibroblasts cells, malignant cells, mast cells, monocytes, myofibroblasts cells and plasma (**Figures 1A–B**). Subsequently, we evaluated the genes that are dominantly expressed in fibroblast cells, which are further regarded as biomarkers for prostate cancer cells. The results demonstrated that COL1A1, TIMP1, MGP, COL1A2, IGFBP7, DCN, BGN, RARRES2, LGALS1 and SPARC are the biomarkers for CAFs (**Figures 1C–L**). The ssGSEA algorithm was then applied to evaluate the distribution of the fibroblasts in different prostate cancer samples. Based on the ssGSEA algorithm, the prostate cancer cohort was divided into low- and high-fibroblasts cell groups (**Figure 1M**).

### Exploration of the differentially expressed genes and the potential pathways involved in the differentially expressed genes

**Figure 2:**
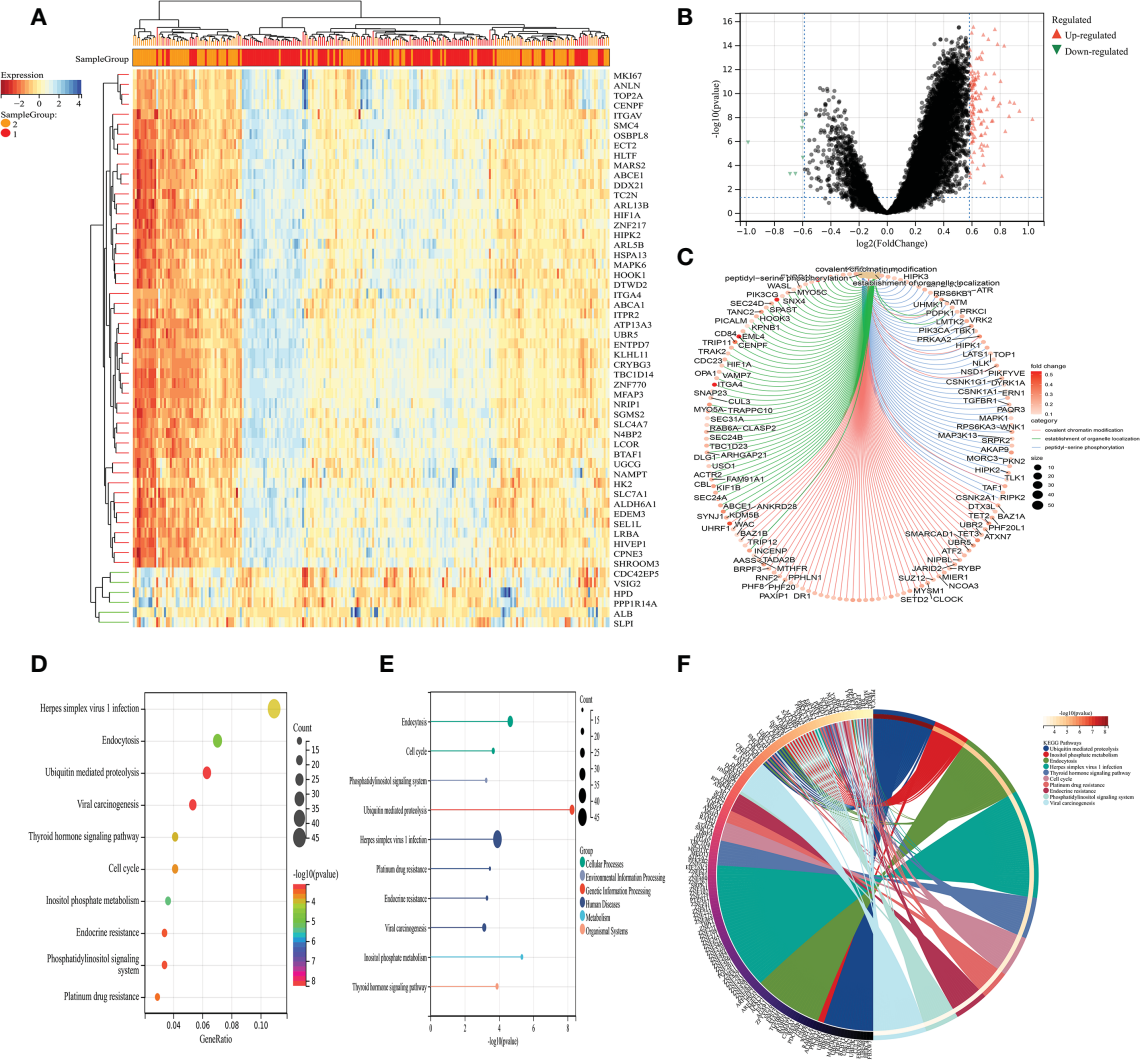
**(A)** The heat map demonstrated the differentially expressed genes between low- and high-CAFs groups; **(B)** The volcano map demonstrated the differentially expressed genes between low- and high-CAFs groups; **(C)** The GO enrichment analysis; **(D-F)** The KEGG enrichment analysis.

Next, on the basis of the previous analysis, the prostate cancer cohort was divided into low- and high-fibroblasts cell groups. Subsequently, we performed the differentially expressed analysis between the low-fibroblasts cells group and the high-fibroblasts cells group. The results demonstrated a total of 127 differentially expressed genes, which consist of 6 down-regulated genes and 121 up-regulated genes (**Figures 2A–B**). Therefore, these 127 genes were regarded as the hub genes that play a key role in the CAFs. Then, the GO and KEGG enrichment analysis was applied to explore the potential pathways that are closely associated with CAFs. The GO enrichment analysis demonstrated that peptidyl-serine phosphorylation, covalent chromatin modification, the establishment of organelle localization, histone modification, peptidyl-serine modification, protein polyubiquitination and protein deubiquitination are the most enriched pathways (**Figure 2C**). For KEGG enrichment analysis, the results revealed that cell cycle, ubiquitin-mediated proteolysis, inositol phosphate metabolism, endocytosis, and endocrine resistance are closely associated with CAFs (**Figures 2D-F**).

### Evaluation of the association between immune-related cells, immune checkpoints-related genes and CAFs

**Figure 3:**
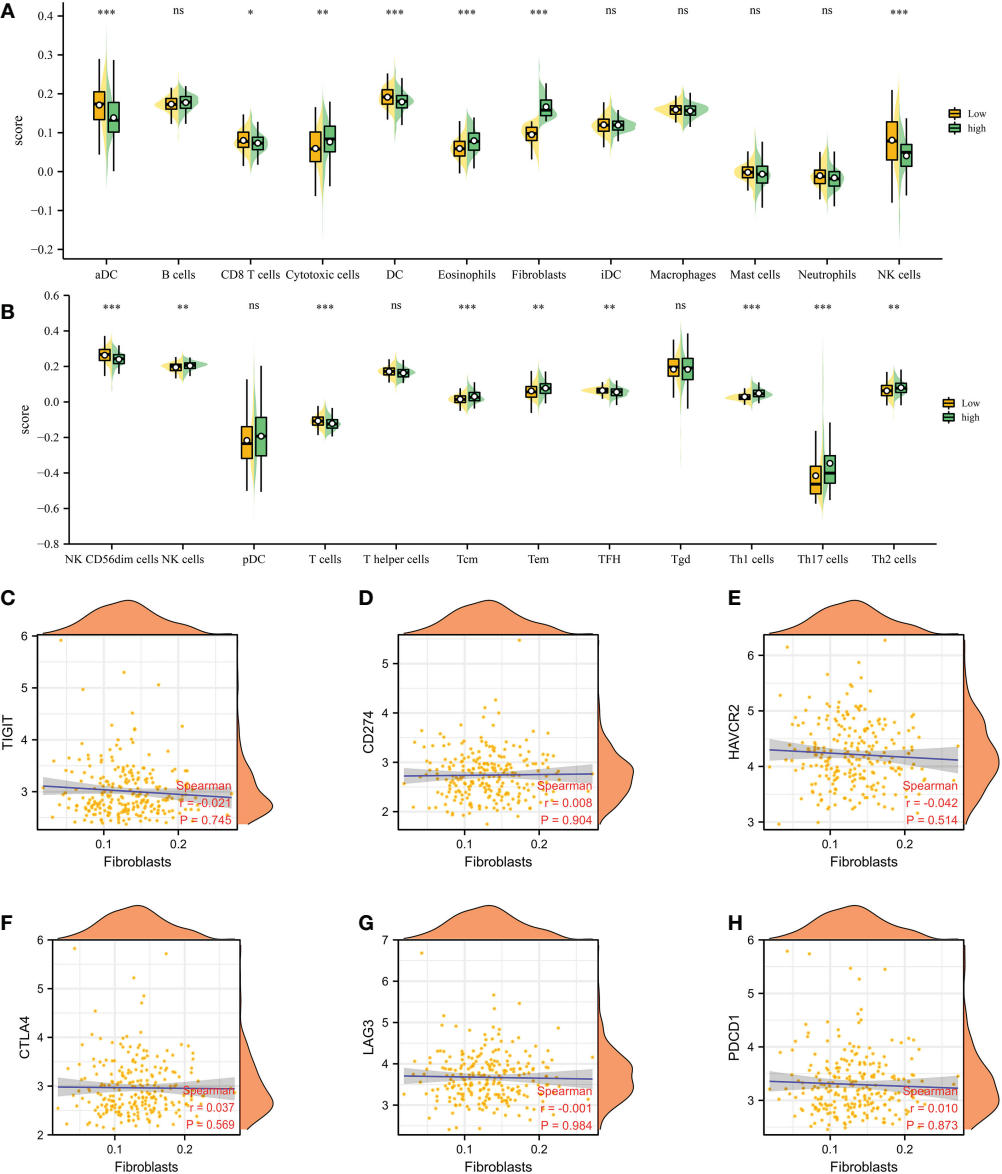
**(A, B)** The immune cell infiltration analysis between low- and high-CAFs groups; **(C-H)** The correlation analysis between fibroblasts and immune checkpoint-related genes. *: p<0.05; **: p<0.01; ***: p<0.001, ns: p>0.05.

Based on the different CAFs scores, we performed the immune cell infiltration analysis. The results demonstrated that many immune-related genes showed different distributions. The results showed that CD8 T cell, cytotoxic cell, DC, eosinophils, fibroblasts, NK cells, NK CD56 dim cells, T cells, Tem, TFH, Tgd, Th1 cells, Th17 cells and Th2 cells are closely associated with the CAFs (**Figuress 3A, B**). Subsequently, we also evaluate the correlation between immune checkpoint-related genes and CAFs. The results showed no significant difference between CAFs and checkpoint-related genes, such as TIGIT, CD274, HAVCR2, CTLA4, LAG3 and PDCD1 (**Figures 3C–H**).

### Construction of the CAFs-related genes-based prognostic prediction model in the prostate cancer cohort

**Figure 4:**
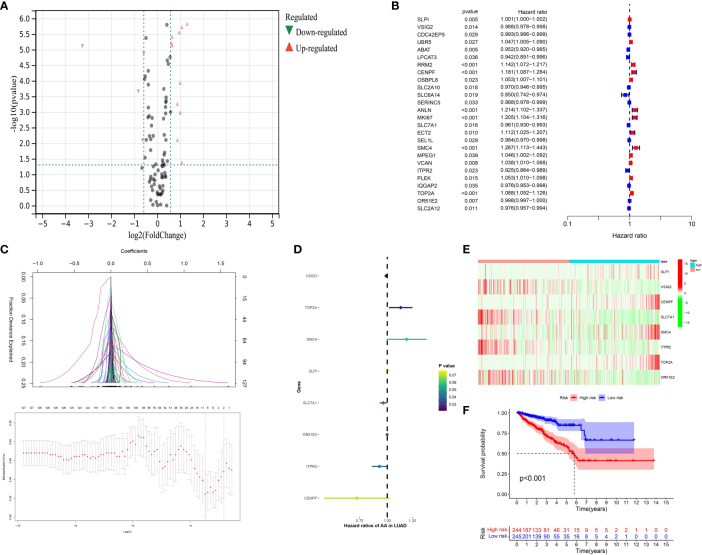
**(A)** The results of the differentially expressed analysis between normal cohort and prostate cancer patients; **(B)** The results of the univariate COX regression analysis; **(C)** The results of the lasso regression analysis based on the prognostic-related genes; **(D)** The results of the multivariate COX regression analysis; **(E)** The heatmap reveals the different group based on the risk score; **(F)** The survival analysis between low- and high-CAFs group.

On the basis of the previous analysis, we successfully obtained a total of 127 hub genes of CAFs. Furthermore, we performed the differentially expressed analysis between a normal cohort and patients with prostate cancer, the results demonstrated there are a total of 3 down-regulated genes and 18 up-regulated genes. (**Figure 4A**) Subsequently, in order to further explore the genes that are closely associated with the Progression-Free-Survival (PFS) of prostate cancer patients, the expression data of 127 hub genes, as well as the clinical information, was merged into a matrix. Then, the univariate COX regression analysis was applied to screen the genes with the prognostic value. The results demonstrated that a total of 26 genes showed good predictive value (**Figure 4B**). The lasso regression analysis and multivariate COX regression analysis were performed to further explore the genes with good prognostic value in patients with prostate cancer (**Figures 4C, D**). The results demonstrated that a total of 8 genes consist of the prognostic prediction model, which includes SLPI, VSIG2, CENPF, SLC7A1, SMC4, ITPR2, TOP2A and OR51E2. The risk score = SLPI* 0.000584811158157081 + VSIG2 * -0.01190627068889 + CENPF * -0.317826812875334 + SLC7A1 * -0.0410213995358753 + SMC4 * 0.202544454923637 + ITPR2 * -0.0824652047622673 + TOP2A * 0.140312081524807 + OR51E2 * -0.00136602095885459. Based on the middle-risk score, the prostate cancer cohort was divided into low-risk and high-risk groups (**Figure 4E**). The survival analysis of PFS demonstrated that patients involved in the high-risk group are associated with poorer PFS (**Figure 4F**).

### Correlation analysis between CAFs-based model and immune cell infiltration, immunotherapy, immune score and clinical characteristics

**Figure 5:**
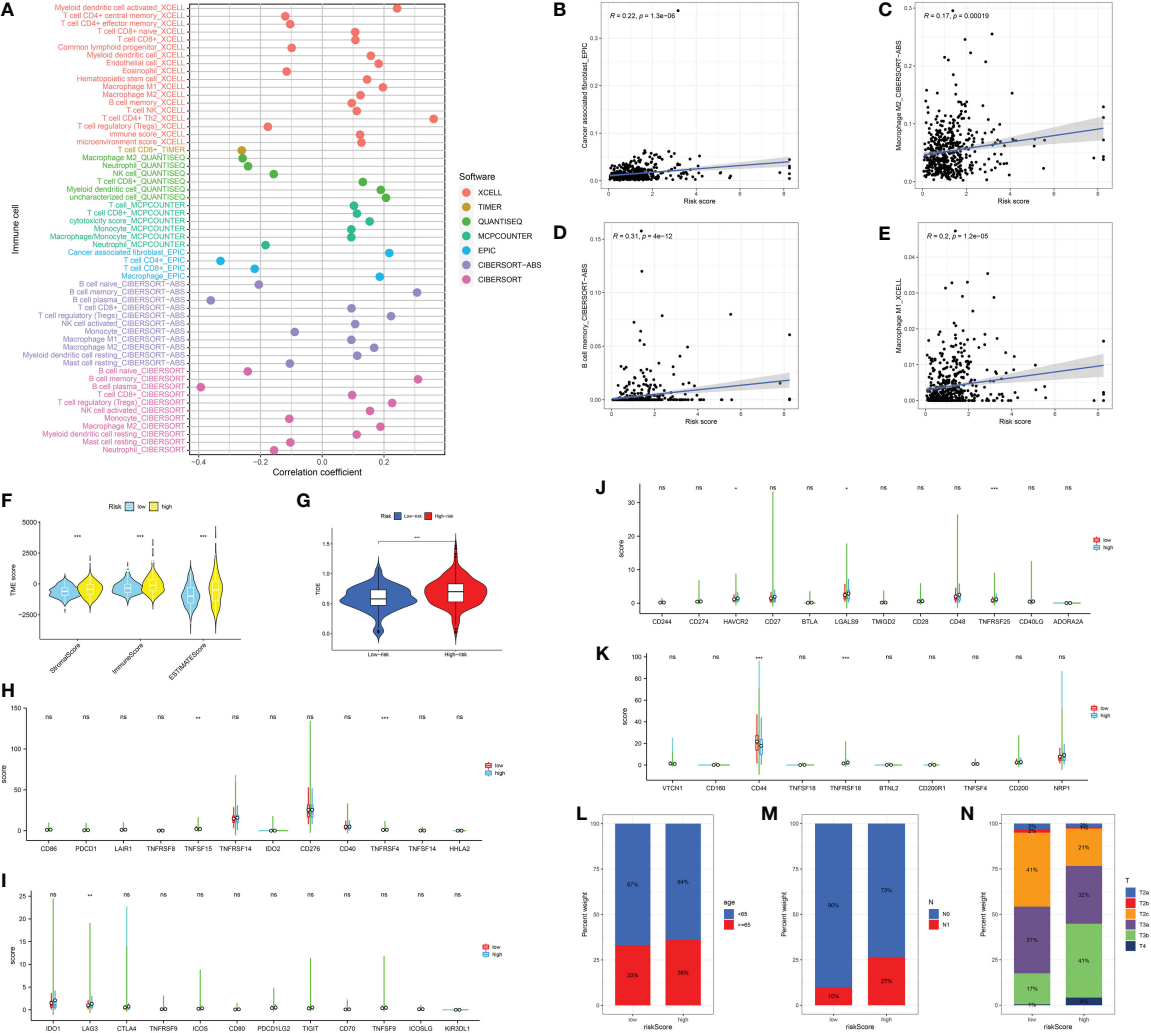
**(A)** The correlation analysis between immune cell infiltration and risk score; The correlation between risk score and CAFs **(B)**; M2 macrophages **(C)**; Memory B cell **(D)**; M1 macrophages **(E)**; **(F)** The association between risk score and immune-related score; **(G)** The correlation analysis between TIDE score and risk score; **(H-K)** The correlation between immune checkpoint-related genes and risk score; The correlation analysis between risk score and age **(L)**; N stage **(M)**; T stage **(N)**. *: p<0.05; **: p<0.01; ***: p<0.001, ns: p>0.05.

Next, in order further explore the value of the CAFs-based model in the immune-related index, we then performed the correlation analysis. In terms of the immune-related cells, the results demonstrated that the risk score is correlated with CAFs, M2 macrophages, memory B cell and M1 macrophages (**Figures 5A–E**). In addition, prostate cancer patients involved in high-risk groups are associated with a higher stromal score, immune score and estimate score (**Figure 5F**). The patients with higher risk score are also associated with the higher TIDE score, which indicates that they may show worse immunotherapy responses (**Figure 5G**). Subsequently, we evaluate the correlation between immune checkpoint-related genes and risk score. The results revealed that TNFRSF15, TNFRSF4, LAG3, HAVCR2, LGALS9, TNFRSF25, CD44, and TNFRSF18 shows the significant difference between low- and high-risk groups (**Figures 5H–K**). In terms of clinical characteristics, patients with higher risk scores are associated with higher age, T stage and N stage (**Figures 5L–N**).

### Exploration of the potential pathways involved in CAFs-based model and hub genes

**Figure 6:**
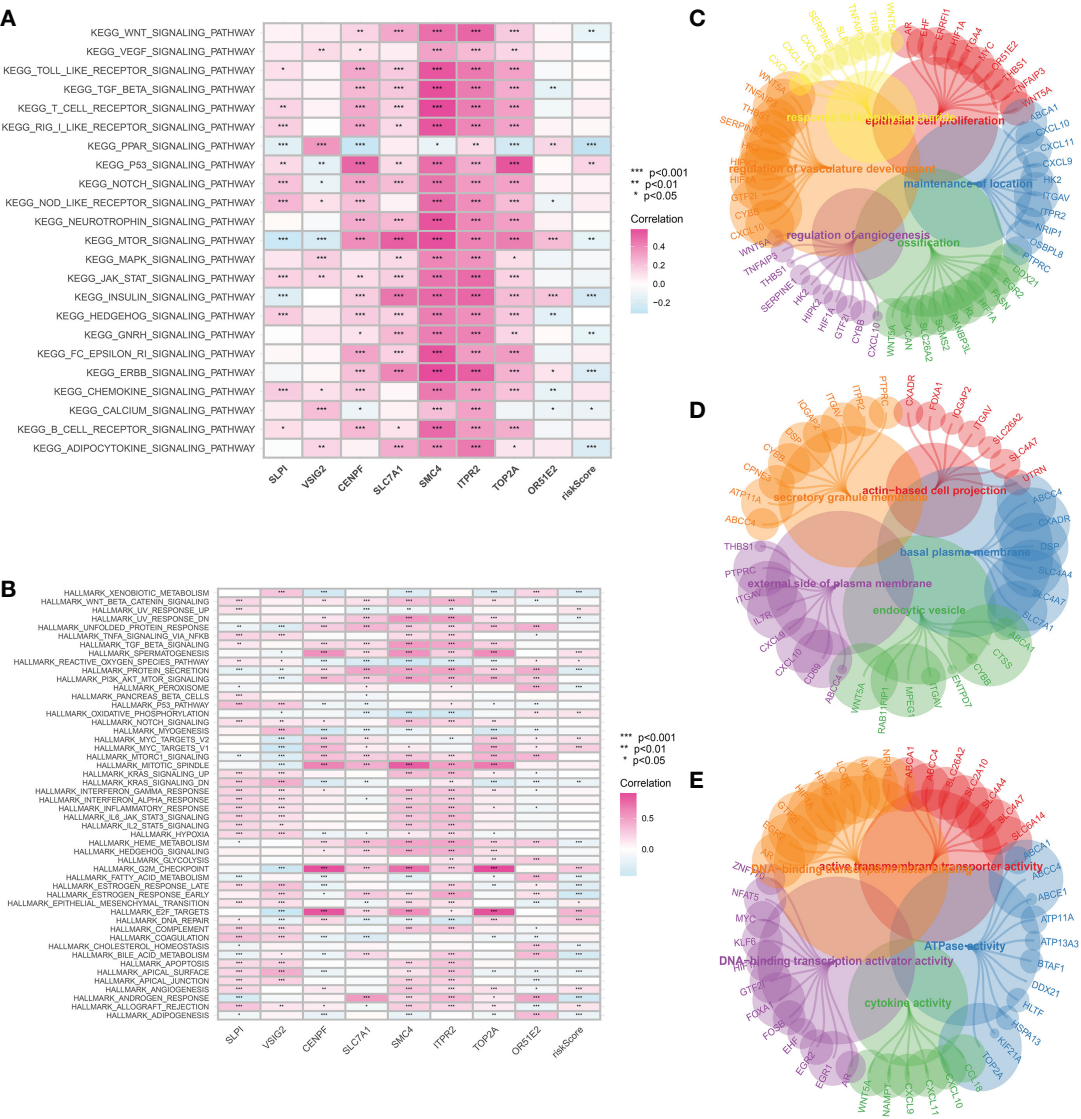
**(A)** The GSVA analysis based on the KEGG terms; **(B)** The GSVA analysis based on the Hallmark terms; The GO enrichment analysis of CAFs-related genes in terms of BP **(C)**, CC **(D)** and MF **(E)**.

The GSVA analysis was then applied to demonstrate the pathways associated with the risk score. In terms of the KEGG terms, adipocytokine signaling pathway, ERBB signaling pathway, GnRH signaling pathway, insulin signaling pathway, mTOR signaling pathway and PPAR signaling pathway are closely associated with the CAFs-based model. For Hallmark terms, the most enriched pathways consist of Xenobiotic metabolism, protein secretion, peroxisome, oxidative phosphorylation and E2F targets (**Figures 6A, B**). The GO enrichment analysis was performed to explore the pathways that are closely associated with hub genes. The GO BP enrichment involves response to lipopolysaccharide, epithelial cell proliferation, regulation of vasculature development, ossification, regulation of angiogenesis and maintenance of the location. The most enriched GO CC enrichment analysis includes actin-based cell projection, secretory granule membrane, basal plasma, external side of plasma membrane and endocytic vesicle. For GO MF, DNA-binding transcription activator activity, ATPase activity and cytokine activity are the most enriched pathways (**Figures 6C–E**).

### CENPF may be the most effective targets involved in the CAFs-based prognostic prediction model

**Figure 7:**
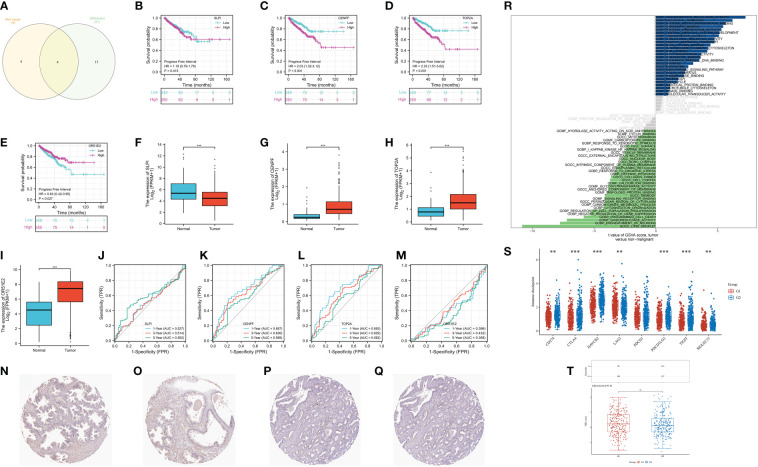
**(A)** The Venn diagram demonstrated the key genes involves in differentially expressed genes and CAFs-related genes; **(B)** The survival analysis of SLPI; **(C)** The survival analysis of CENPF; **(D)** The survival analysis of TOP2A; **(E)** The survival analysis of OR51E2; **(F)** The expression of SLPI between normal cohort and prostate cancer cohort; **(G)** The expression of CENPF between normal cohort and prostate cancer cohort; **(H)** The expression of TOP2A between normal cohort and prostate cancer cohort; **(I)** The expression of OR51E2 between normal cohort and prostate cancer cohort; **(J)** The time-dependent ROC curve of SLPI; **(K)** The time-dependent ROC curve of CENPF; **(L)** The time-dependent ROC curve of TOP2A; **(M)** The time-dependent ROC curve of OR51E2; **(N, O)** The expression of CENPF protein in normal prostate; **(P, Q)** The expression of CENPF protein in prostate cancer tissues; **(R)** The GSVA analysis of CENPF in prostate cancer cohort; **(S)** The expression level of immune checkpoint-related genes between low and high-expression level of CENPF group; **(T)** The TIDE score between low- and high- expression level of CENPF group. **: p<0.01; ***: p<0.001, ns: p>0.05.

Finally, we aim to find the gene that plays the role in the CAFs-based prognostic prediction model. The Venn diagram demonstrated that four genes are involved in differentially expressed genes and CAFs-based model, including SLPI, CENPF, TOP2A and OR51E2 (**Figure 7A**). The survival analysis revealed that progress-free interval is closely associated with the expression level of CENPF, TOP2A, and OR51E2 in the prostate cancer cohort (**Figures 7B–E**). The boxplot demonstrated that SLPI is down-regulated in prostate cancer patients, while the CENPF, TOP2A and OR51E2 are up-regulated in prostate cancer patients compared with the normal cohort (**Figures 7F–I**). The time-dependent ROC curve demonstrated that CENPF and TOP2A show good predictive value in prostate cancer patients (**Figures 7J–M**). Additionally, the protein expression level of CENPF in prostate cancer is high than in normal prostate tissues (**Figures 7N–Q**). The GSVA analysis revealed that CENPF upregulates the pathways of transcription regulator activity, biological adhesion, histone methyltransferase binding and synapse, and downregulates the pathways of lipid droplet, ensheathment of neurons, oxidoreductase activity and programmed cell death (**Figure 7R**). The immune checkpoint-related genes (CD274, CTLA4, HAVCR2, LAG3, PDCD1LG2, TIGIT and SIGLEC15) showed a significant difference between low- and high-expression of the CENPF group (**Figure 7S**). However, the TIDE score showed no difference between the different expression levels of CENPF (**Figure 7T**).

### The CENPF can promote the proliferation ability of prostate cancer cells

**Figure 8:**
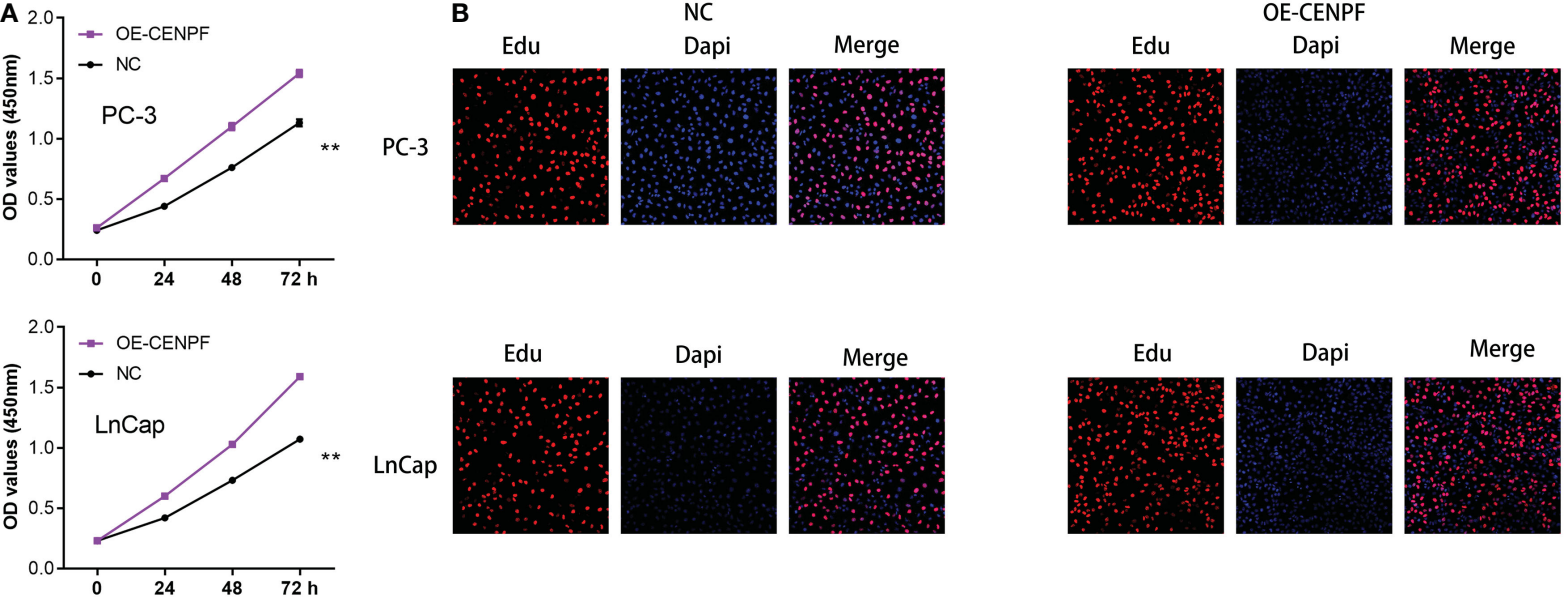
**(A)** The CCK8 assay demonstrated the proliferation ability in prostate cancer cells; **(B)** The Edu assay demonstrated the proliferation ability in prostate cancer cells. **: p<0.01.

On the basis of the previous studies, we discovered that CENPF may play a key role in the progression of prostate cancer. Therefore, we aim to further explore the role of CENPF in prostate cancer cells. The CCK8 assay demonstrated that the proliferative ability of PC-3 and LNCap cells was improved with the overexpressed of CENPF compared with an empty vector (**Figure 8A**). In addition, the Edu staining assay also proved that the proliferation ability of prostate cancer cells was also enhanced with the overexpressed of CENPF (**Figure 8B**).

## Discussion

It is estimated that 248,530 cases of prostate cancer will be diagnosed in the United States in 2021, making it the most common cancer among men (18). A projected 34,130 men died from this disease in the United States in 2021, making it the second leading cause of death among men (19). Patients with prostate cancer can be diagnosed with prostate cancer using serum prostate-specific antigen (PSA) (20). However, it has been shown to lack specificity, which can lead to prostate cancer being overdiagnosed and overtreated. Therefore, it is urgent to explore the potential biomarkers for better diagnosis and treatment of patients with prostate cancer. The tumor microenvironment has received increased attention due to its critical role in tumor immunosuppression, distant metastasis, local drug resistance, and response to targeted therapies (21). Researchers are currently focusing more and more on CAFs’ immunosuppressive properties, which are achieved through the interaction with temporary components, including immune cells (22). It has been demonstrated that CAFs not only promotes tumor proliferation, but they also induce immune evasion and provide structural support for angiogenesis and invasion of tumors (23). Further research is needed to clarify the specific roles and detailed mechanisms of CAFs in cancer pathogenesis and progression (24). In recent years, many studies begin to apply bioinformatics analysis to explore the potential biomarkers for better diagnosis and more suitable treatment of prostate cancer patients (25, 26). In this work, we aim to explore the potential associations between CAFs and prostate cancer and figure out the role of immunotherapy in CAFs.

First of all, with the help of single-cell RNA sequencing, we obtained the biomarkers of fibroblasts in prostate cancer. The COL1A1, TIMP1, MGP, COL1A2, IGFBP7, DCN, BGN, RARRES2, LGALS1 and SPARC were dominantly expressed in the fibroblasts, which were regarded as the biomarkers for fibroblasts. By using the ssGSEA algorithm, the prostate cancer cohort was divided into different subtypes based on the different distributions of CAFs. The immune cell infiltration analysis revealed the close association between the CAFs and immune-related cells. Besides promoting tumor initiation and progression, CAFs also shape the microenvironment of tumors. Cancer-associated factors (CAFs) interact with tumor cells either by cell-to-cell contact or by releasing soluble factors into the tumor environment (27). Further, CAFs cross-talk actively with other tumor microenvironmental members in addition to this interaction. Furthermore, various recent studies have shown that CAFs can modulate the function and recruitment of immune cells (28). According to a recent study, CAFs isolated from invasive breast cancer can induce the differentiation of monocytes into M2 macrophages, compared with normal breast-derived fibroblasts (29). By inducing immunosuppressive macrophages, CAF may contribute to the establishment of an immune-suppressive environment.

Furthermore, we also performed the enrichment pathway analysis, the results demonstrated that CAFs involve lots of pathways, such as cell circle and endocrine resistance. The primary purpose of cancer-associated mutations that disrupt cell cycle control is to enable continuous cell division by impairing the ability of the cell to exit the cycle. Cell cycle control mechanisms and their role in cancer are now known to us in greater detail, revealing how these dependencies can best be exploited in cancer treatment (30). Lots of the studies have covered the close association between androgen and prostate cancer. The first-line treatment for advanced prostate cancer involves androgen deprivation therapy (31). The treatment of androgen deprivation works well for most patients. However, over time, androgen deprivation therapy develops resistance and patients become desensitized to androgen withdrawal or androgen receptor inhibition (32). The results of enrichment analysis revealed that CAFs may influence the process of prostate cancer by interfering with the cell cycle and endocrine resistance.

Subsequently, in order to evaluate the prognostic value of CAFs in prostate cancer patients, we then construct the prognostic model. The CAFs-based prognostic model is highly correlated with immune cell infiltration, immune score, immunotherapy and immune checkpoint-related genes. In addition, the patients involved in the high-risk group are associated with the higher T and N stage. Compared to nonmalignant prostate fibroblasts, CAFs promote tumor growth in prostate cancer. It was previously shown that CAF-derived LOXL2 plays an important role in intercellular communication in the prostate tumor microenvironment and could be used to treat prostate cancer (33). Further analysis demonstrated that CENPF may be the most effective target involved in the CAFs-based prognostic prediction model. The differentially expressed analysis revealed that CENPF is the significantly higher expression in prostate cancer patients, and is associated with the poor progress-free interval of prostate cancer patients. In addition, the time-dependent ROC curve also proves the good predictive value of CENPF in prostate cancer. Finally, we performed the cell proliferation assays based on the prostate cancer cells. The results demonstrated that the overexpressed of the CENPF could promote the ability of proliferation in prostate cancer cells. A former study demonstrated that CENPF silencing alters the metabolic profile of prostate cancer cells and inhibits cell proliferation, suggesting that CENPF may be a key regulator of prostate cancer metabolism (33).

However, there are some limitations in the analysis based on the online dataset. First, the dataset involved in this manuscript was mainly from the TCGA dataset, which may lead to bias (34). In addition, the limited samples of prostate cancer patients from the TCGA dataset may lead to different results of analysis (35). Therefore, it is important to perform further analysis to obtain the results more precisely (36) In conclusion, we discuss the potential prognostic and therapeutic value of CAF-dependent pathways in prostate cancer. In addition, CENPF may be a promising CAF-dependent biomarker for the diagnosis and prognosis of prostate cancer.

## Data availability statement

The original contributions presented in the study are included in the article/supplementary material. Further inquiries can be directed to the corresponding authors.

## Author contributions

XC, ZW, XZ, XC and MT collected the data and performed the analysis. MG, YD and JG wrote the manuscript. All authors contributed to the article and approved the submitted version.
